# Disseminated nontuberculous mycobacterial infection in a patient with idiopathic CD4 lymphocytopenia and IFN-γ neutralizing antibodies: a case report

**DOI:** 10.1186/s12879-023-08020-6

**Published:** 2023-01-30

**Authors:** Ryo Goto, Seiji Shiota, Ryo Kaimori, Noboru Horinouchi, Rie Utsunomiya Nishimizu, Kyoko Yamamoto, Eishi Miyazaki

**Affiliations:** Department of General Medicine, Oita University Faculty of Medicine, 1-1 Idaigaoka, Hasama-Machi, Yufu, Oita 879-5593 Japan

**Keywords:** Disseminated nontuberculous mycobacterium (NTM) infection, Anti-interferon-γ autoantibodies, CD4 lymphocytopenia, Case report

## Abstract

**Background:**

Disseminated nontuberculous mycobacterial (NTM) infection usually occurs in immunodeficient patients, such as those with human immunodeficiency virus infection and idiopathic CD4 lymphopenia. However, disseminated NTM diseases have also been reported in immunocompetent patients. Autoantibodies to interferon-gamma (IFN-γ) are known to be involved in disseminated NTM disease, although anti-IFN-γ antibodies are mainly seen in immunocompetent patients rather than those with immunodeficiency. Here, we report a rare case of disseminated NTM patient with idiopathic CD4 lymphopenia and anti-IFN-γ antibodies.

**Case presentation:**

A 64-year-old Asian male presented with fever, back pain, anorexia and weight loss. Physical examination revealed subcutaneous masses in the forehead, sternoclavicular joint, and right inguinal region. Computed tomography showed multiple osteosclerotic changes with soft structures and osteolytic changes. Both blood and sputum cultures were positive for *Mycobacterium intracellulare,* confirming the presence of disseminated NTM infection. Histopathological evaluation of the subcutaneous mass in the right inguinal region showed numerous granulomas consisting of epithelioid cells with Langhans-type giant cells. He was diagnosed with idiopathic CD4 lymphocytopenia. Interestingly, he also had anti-IFN-γ autoantibodies with suppression of IFN-γ-dependent signal transducer and activator of transcription 1 (STAT1) phosphorylation. Two-drug combination therapy with clarithromycin and ethambutol was started for the NTM infection, which resulted in a favorable disease course.

**Conclusions:**

In patients with disseminated NTM infection, idiopathic CD4 lymphocytopenia and anti-IFN-γ autoantibody-positive immunodeficiency can be coexisted. It is necessary to clarify the pathogenesis and clinical course of CD4 lymphocytopenic conditions and IFN-γ neutralizing antibody-positive in the disseminated NTM disease.

## Background

Disseminated nontuberculous mycobacterial (NTM) infection is defined by localized growth of mycobacteria, with subsequent invasion of other organs and tissues via the hematological route [1]. The symptoms are typically nonspecific, with intermittent or persistent fever, night sweats, weight loss, malaise, and anorexia. Diagnosis is made by detection of Mycobacterium on blood culture. Culture and histopathology of bone marrow biopsy specimens and fluid or tissue from the suspected site of infection also aid in the diagnosis.

Disseminated NTM infection is traditionally thought to occur in immunodeficient persons, such as those with human immunodeficiency virus (HIV) infection, on steroid therapy, or malignant tumors [1, 2]. In addition, disseminated NTM infection is occur in the patients with idiopathic CD4 lymphocytopenia, which absolute CD4 T lymphocyte count of less than 300 cells/μL [2]. Interferon-gamma (IFN-γ), which is an activator of macrophage differentiation and a proinflammatory activator of innate immunity, is supposed to play a crucial role in disseminated NTM infection [2]. Some cases of disseminated NTM disease have been reported in the absence of known immunodeficiency, and anti-IFN-γ autoantibody that neutralize IFN-γ are reported to play a role in their pathogenesis [3]. Neutralizing capacity for IFN-γ was thought to be more important than the antibody concentration itself in disseminated NTM infection [4]. While the positivity rate of neutralizing antibodies to IFN-γ is reportedly high in immunocompetent patients with disseminated NTM infection, the antibodies are rarely seen in immunocompromised patients [4]. Here, we report a case of disseminated NTM infection in a patient with neutralizing antibodies to IFN-γ, in addition to idiopathic CD4 lymphocytopenia, but with no previous immunodeficiency.

## Case presentation

A 64-year-old male visited a previous hospital complaining of chest and back pain that progressively worsened over a period of 2 months, fever, anorexia, weight loss of about 10 kg, and dyspnea. His symptoms did not improve despite antibiotic treatment with tazobactam/piperacillin (TAZ/PIPC) for suspected bacterial pneumonia. Bone marrow biopsy from the hip bone, which was performed because of elevated serum soluble interleukin-2 receptor (sIL-2R) levels, showed the presence of many epithelioid cell granulomas with Langhans-type giant cells. The patient was transferred to our hospital for further examination. Seven years earlier, he had been suspected to have malignant lymphoma based on the presence of malaise, fever, enlarged lymph nodes, etc., although bone marrow biopsy and other tests had failed to reveal a diagnosis. CD4 levels were not examined at that point. The patient had smoked 15 cigarettes/day since the age of 24 years, although he only occasionally drank alcoholic beverages.

**Fig. 1:**
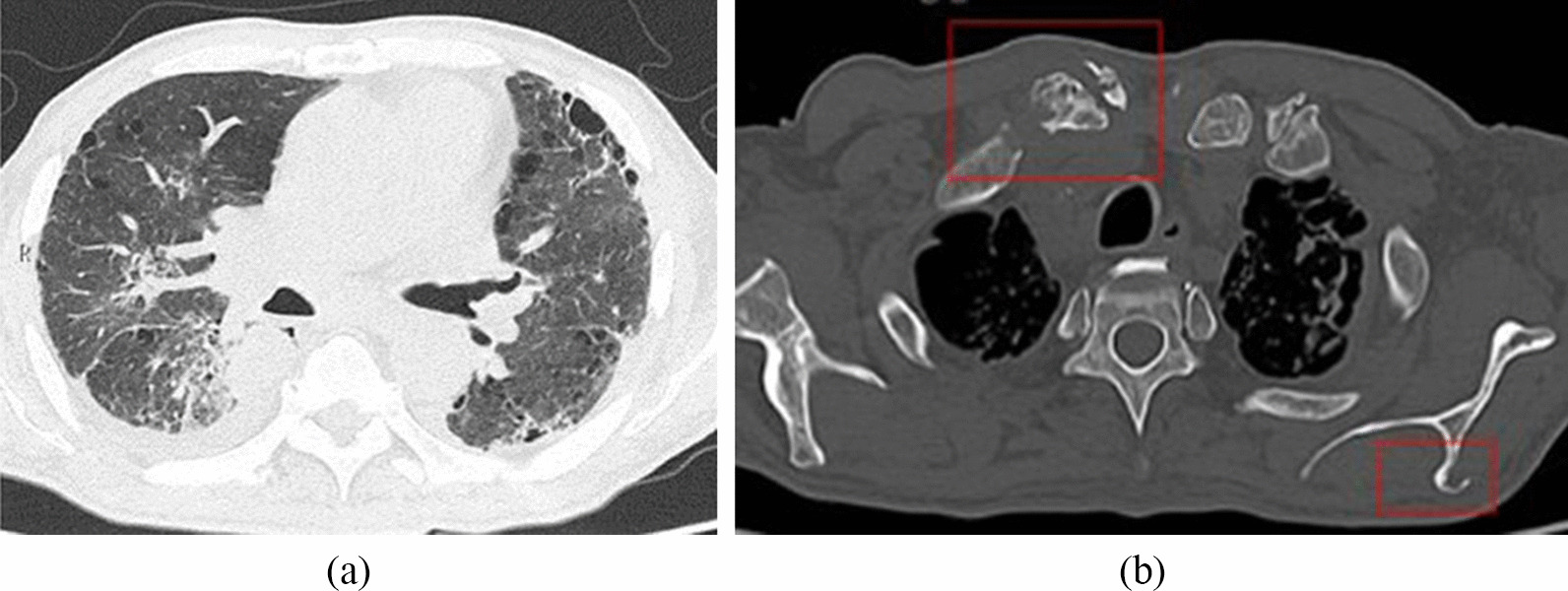
Chest computed tomography scans showed a mass in the right S6 lung region. Pleural effusion and emphysematous changes were also observed (**a**). Osteolytic changes were observed in the right clavicle and left scapula (**b**)

**Fig. 2:**
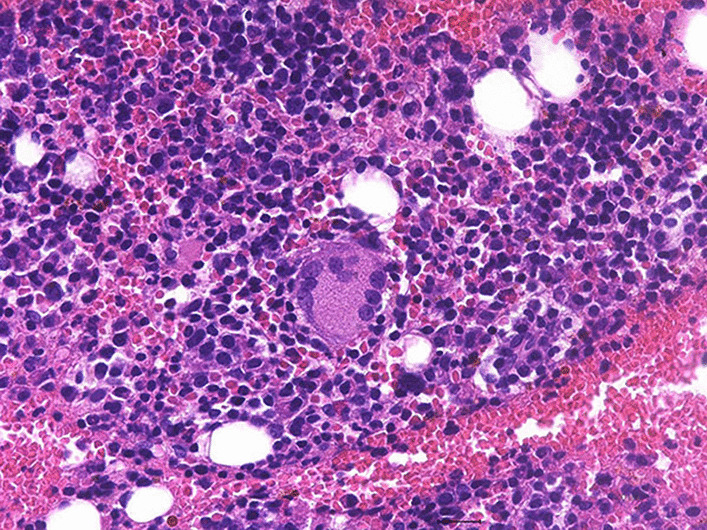
Histopathological evaluation of tissue obtained from a subcutaneous mass in the right inguinal region showed numerous granulomas consisting of epithelioid cells with Langhans-type giant cells

Physically, he was 161.0 cm tall and his body weight was 61.7 kg after a 10 kg weight loss over the previous one month. His vital signs were: temperature 36.7 °C, blood pressure 126/69 mmHg, pulse rate 103 beats/min, and oxygen saturation 96% while breathing 2 L/min oxygen via a nasal cannula. Physical examination revealed no abnormal heart or respiratory sounds. Palpation revealed no abnormalities in the abdomen. However, non-tender subcutaneous masses (approximately 20 mm × 20 mm) were noted in the forehead, sternoclavicular joint and right inguinal region. Blood tests showed: albumin 1.92 g/dL, Na 137.6 mEq/L, K 2.91 mEq/L, corrected Ca 12.89 mg/dL, C-reactive protein (CRP) 10.79 mg/dL, and ferritin 399.8 ng/mL (Table 1). There were no abnormalities in liver or kidney function. Urinalysis showed no abnormal findings. Levels of leukocytes were 6820/μL (neutrophils 6070/μL, lymphocytes 380/μL), CD4 + were 107/μL, and CD8 + were 64/μL. Immunoglobulin levels were elevated (IgG 2765 mg/dL, IgM 334.2 mg/dL, and IgA 565 mg/dL), while those of complement were decreased (C3 60 [normal: 73–138 mg/dL], C4 3.7 [normal: 11–31 mg/dL], and CH50 20.4 [normal: 31.6–57.6 U/mL]). The level of sIL-2R was elevated to 7829 IU/mL. Anti-human immunodeficiency virus (HIV) antibody and anti-human T cell leukemia virus (HTLV)-1 antibody were negative. He was diagnosed with idiopathic CD4 lymphocytopenia, since the CD4 + and CD8 + fractions measured over time after admission were below normal at all time points, and he was negative for HIV and HTLV-1 antibodies and had no underlying diseases or history of drug usage to explain the immunodeficiency. Whole blood IFN-γ release assay (using QuantiFERON TB-3G [QFT]) showed a low IFN-γ level even with mitogen (Phytohemagglutinin [PHA]) stimulation. Serum anti-MAC antibodies were positive. Chest computed tomography (CT) showed diffuse emphysematous changes in bilateral chest areas, increased parenchymal density, destruction of alveolar structures, and bronchial wall thickening (Fig. 1a). It also showed infiltration in the right lower lung lobes. CT also revealed overall diffuse heterogeneous osteosclerosis in the bones, with soft-tissue structures around the acromioclavicular and sternoclavicular joints. Numerous other osteolytic changes were observed in the ribs, clavicle, scapula, ilium, right sciatic bone, and bilateral femoral heads (Fig. 1b). Both blood and sputum cultures were positive for *Mycobacterium intracellulare*. Histopathological evaluation of the subcutaneous mass in the right inguinal region showed numerous granulomas consisting of epithelioid cells with Langhans-type giant cells (Fig. 2). These findings confirmed the diagnosis of disseminated NTM infection. Hence, two-drug combination therapy with clarithromycin (CAM) and ethambutol (EB) was started. Although rifampicin was added, it was withdrawn due to pancytopenia. The CD4 + and CD8 + fractions did not improve in spite of improvement in clinical symptoms. Subsequently, we evaluated the neutralizing activity of his anti-IFN-γ autoantibodies using signal transducer and activator of transcription 1 (STAT1) phosphorylation in whole blood leukocytes with IFN-γ stimulation, as described previously [5]. The anti-IFN-γ autoantibodies detected in our patient clearly suppressed IFN-γ-dependent STAT1 phosphorylation, which confirmed the diagnosis of anti-IFN-γ autoantibody-positive immunodeficiency. The patient was transferred to the referring hospital on the 62nd day of hospitalization. He is currently on a combination of CAM and EB and has been relapse-free for 1 year.

**Table 1:** Laboratory findings

WBC (/μL)	6820	TP (g/dL)	7.27	IgG (mg/dL)	2765
Neu	6070	Alb (g/dL)	1.92	IgA (mg/dL)	565
Mon	190	T-bil (mg/dL)	0.45	IgM (mg/dL)	334.2
Lym	330	AST (U/L)	10.3	C3 (mg/dL)	60
Eos	190	ALT (U/L)	5.2	C4 (mg/dL)	3.7
Bas	40	LD (U/L)	107	CH50 (U/mL)	20.4
RBC (/μL)	2.89 × 10^6^	γ-GTP (U/L)	28.1	β-D glucan (pg/mL)	< 3.499
Hb (/dL)	8.9	CK (U/L)	9.0	HBs-Ag	(–)
Plt (/μL)	268 × 10^3^	BUN (mg/dL)	13.6	HCV-Ab	(–)
ESR (mm/h)	> 120	Cre (mg/dL)	0.8	HIV-Ab	0.1
		Na (mEq/L)	137.6	HTLV-1-Ab	0
		K (mEq/L)	2.91	sIL2-R (U/mL)	7829
		Cl (mEq/L)	95.9	CD4 (/μL)	107
		Corrected Ca (mg/dL)	12.89	CD8 (/μL)	64
		HbA1c (%)	5.0%		
		CRP (mg/dL)	10.79		
		Ferittin (ng/mL)	399.8		

*sIL-2R* soluble IL-2 receptor

## Discussion and conclusions

Disseminated NTM infection is occur in immunodeficient persons, such as those with HIV infection and idiopathic CD4 lymphocytopenia. Idiopathic CD4 lymphopenia is defined as a CD4 T-lymphocyte count of less than 300 cells/μL or less than 20% of total T-cells on more than one occasion, in the absence of HIV infection and any other immunodeficiency or therapy associated with depressed levels of CD4 T-cells [2]. Since our patient continuously showed decreased CD4 + levels and had no history of congenital immunodeficiency or acquired lymphopenia, we diagnosed idiopathic CD4 lymphocytopenia, although it was not clear when the onset of the disease occurred. In general, patients with idiopathic CD4 lymphocytopenia develop several diseases, such as cryptococcal disease, persistent genital human papilloma virus (HPV) infection, and NTM infection [6]. Our patient did not develop these conditions until his current presentation.

When invading bacteria are phagocytosed by macrophages, IL-12 is secreted, which stimulates T lymphocytes. The stimulated T lymphocytes secrete IFN-γ, which activates phagocytic cells to eliminate the bacteria. The IFN-γ-IL-12 axis plays an important role in the defense against intracellular parasites such as acid-fast bacilli [7]. IFN-γ is thought to be important for infection control in disseminated NTM infection. In fact, in a previous report, IFN-γ was used to treat a patient with disseminated NTM disease [2]. In some patients, autoantibodies to IFN-γ, which neutralized IFN-γ activity are known to be involved in disseminated NTM disease [3], and the immunodeficiency caused by this disease is called anti-IFN-γ autoantibody-positive immunodeficiency. Among anti-IFN-γ antibodies, the presence of antibodies that neutralize IFN-γ is important for the diagnosis of anti-IFN-γ autoantibody-positive immunodeficiency [3]. In fact, anti-IFN-γ antibodies found in patients with disseminated NTM also have neutralizing activity.[3, 4] On the other hand, anti-IFN-γ antibodies found in patients without disseminated NTM do not have neutralizing activity [3, 4]. In the present patient, IFN-γ-dependent phosphorylation of STAT1 was inhibited, indicating that his anti-IFN-γ antibodies had neutralizing activity. Furthermore, QFT showed a low IFN-γ level even with PHA stimulation in our case. In immunodeficient patients with anti-IFN-γ autoantibodies, IFN-γ secreted by PHA stimulation is neutralized by the antibodies and is thus undetectable, which was one of the reasons to suspect the presence of anti-IFN-γ autoantibody-positive immunodeficiency in our patient.

Interestingly, our patient had not only idiopathic CD4 lymphocytopenia, but was also anti-IFN-γ neutralizing antibody-positive immunodeficiency. To the best of our knowledge, the coexistence of anti-IFN-γ autoantibody-positive immunodeficiency and idiopathic CD4 lymphocytopenia has not been previously reported. This might be considered as evidence that the presence of anti-IFN-γ neutralizing antibodies should only be investigated in cases with no obvious immunodeficiency, such as due to HIV infection or idiopathic CD4 lymphocytopenia. Aoki et al. reported that among patients with disseminated NTM disease, anti-IFN-γ autoantibodies were detected in 81.1% (30 of 37 patients) of immunocompetent patients, but in only 7.8% (1 of 13 patients) of immunodeficient patients [4]. However, the details of the immunocompromised patients were not stated in their paper. Further, there are no previous reports on the clinical or therapeutic course of disseminated NTM disease in patients with anti-IFN-γ neutralizing antibody-positive immunodeficiency and idiopathic CD4 lymphocytopenia. Our patient responded well to antimicrobial agents and has not relapsed over one year of observation with continuous administration of two antimicrobial agents. However, little is known about the pathogenesis and clinical course of IFN-γ neutralizing antibody-positive disseminated NTM in CD4 lymphocytopenic conditions, including HIV and idiopathic CD4 lymphocytopenia, and further accumulation of cases is needed to elucidate the pathophysiology of anti-IFN-γ neutralizing antibodies in idiopathic CD4 lymphocytopenia.

Many differences in disseminated NTM infection have been reported between cases that are anti-IFN-γ neutralizing antibody-positive and those that are antibody negative. Most cases of anti-IFN-γ antibody-positive disseminated NTM disease were reported in Asians [3]. Aoki et al. compared disseminated NTM manifestations between subjects with (n = 31) and without (n = 19) anti-IFN-γ neutralizing antibody [4]. Median of age at disseminated NTM infection onset was higher in anti-IFN-γ neutralizing antibody-positive patients than those in antibody-negative patients (66 vs. 51 years old). In addition, leukocyte levels at onset were higher in anti-IFN-γ neutralizing antibody-positive patients than those in antibody-negative patients (12,010 vs. 6700 cells/μL). CRP also higher in anti-IFN-γ neutralizing antibody-positive patients than those in antibody-negative patients (6.6 vs. 4.6 mg/dL). There was no significant difference in NTM species in subjects with and without anti-IFN-γ neutralizing antibody. Further, the prognosis was reported to be significantly better in disseminated NTM patients with anti-IFN-γ neutralizing antibodies than those who were antibody-negative (mortality 3.2% vs. 15.7%) [4].

In previous disseminated NTM cases with anti-IFN-γ neutralizing antibodies, the patients’ antibody titers remained positive for a long period of time [4]. Further, lifetime antimicrobial therapy is important in disseminated NTM infections, because most patients who stop taking antimicrobials relapse [4]. In other words, patients with anti-IFN-γ neutralizing antibodies are at persistent risk for NTM. In a previous report, B-cell targeted therapy with rituximab, an anti-CD20 monoclonal antibody, to inhibit IFN-γ antibody production was reported to be effective [8]. Another previous case report showed that daratumumab targeting CD38, which is highly expressed on plasma cells and early mature B cells, was effective in reducing IFN-γ autoantibody titers and improving clinical symptoms in a patient with anti-IFN-γ neutralizing antibody-positive immunodeficiency disease [9]. This suggests that for disseminated NTM with concomitant immunodeficiency, as in the present case, it might be desirable to consider treatment against the anti-IFN-γ neutralizing antibody itself.

In conclusion, we report an unusual case of disseminated NTM disease with both anti-IFN-γ antibody positivity and idiopathic CD4 lymphocytopenia, in the absence of innate immunodeficiency. It is necessary to clarify the pathogenesis and clinical course of CD4 lymphocytopenic conditions and IFN-γ neutralizing antibody-positive in the disseminated NTM disease.

## Data Availability

The datasets used during the current study are available from the corresponding author on reasonable request.

## Abbreviations

CAM: Clarithromycin
CRP: C-reactive protein
EB: Ethambutol
HIV: Human immunodeficiency virus
HPV: Human papilloma virus
HTLV: Human T cell leukemia virus
IFN-γ: Interferon-gamma
IL-12: Interleukin-12
NTM: Nontuberculous mycobacterium
PHA: Phytohemagglutinin
QFT: QuantiFERON TB-3G
STAT1: Signal transducer and activator of transcription 1
TAZ/PIPC: Tazobactam/piperacillin
sIL-2R: Soluble interleukin-2 receptor
